# Alcohol Dependence and Rib Fracture Outcomes: A Systematic Review and Meta-Analysis

**DOI:** 10.7759/cureus.42639

**Published:** 2023-07-29

**Authors:** Fiona Field, Jenny Olsson, Anna Hurley

**Affiliations:** 1 Trauma Sciences, Queen Mary University of London, London, GBR; 2 Anaesthetics, King's College Hospital NHS Foundation Trust, London, GBR; 3 Plastic Surgery, Hull University Teaching Hospitals, Hull, GBR

**Keywords:** alcohol disorder, trauma outcomes, blunt thoracic trauma, alcohol dependence, rib fractures

## Abstract

Chronic alcohol use has been associated with impaired pulmonary function, increased risk of pneumonia and poor outcomes after trauma. With a high incidence of rib fractures in this population, the clinical and physiological factors associated with alcohol dependence may influence how these patients recover from thoracic injuries. Therefore, the aim of the systematic review was to examine the effect of alcohol dependence on rib fracture outcomes. The Embase, PubMed and Web of Science databases were searched for studies examining adult patients with rib fractures, with and without a history of alcohol dependency. The outcomes of interest were mortality, pulmonary complications, intensive care length of stay, ventilator days and hospital length of stay. A meta-analysis was performed to combine the data and compare results. Three studies met the criteria for inclusion in the review and all studies were observational in design. Alcohol dependency was associated with increased mortality (OR 1.44 (95% CI: 1.33-1.56)), pneumonia (OR 2.14 (2.02-2.27)) and ARDS (OR 1.71(1.48-1.98)) as well as longer stays in hospital and intensive care (p<0.05). No difference was found in ventilator days between the two groups. Early intensive care review should be considered to reduce complications in this population alongside prompt management of withdrawal symptoms. However, limited primary research exists on this topic and the quality of current evidence is low. Additional primary research is needed to further understand this correlation and draw meaningful conclusions.

## Introduction and background

Alcohol abuse is accountable for 5.3% of all deaths worldwide and carries a significant risk of injury to both individuals and society [1]. It has been associated with poor outcomes following trauma, with evidence that impaired immunological and neurological function may be responsible [2,3]. Rib fractures are a particularly common injury in this patient population and have been attributed to the prevalence of violence, road traffic accidents and reckless behaviour whilst intoxicated [4].

There is a high risk of pulmonary complications after rib fractures due to the effects of direct lung injury and altered lung mechanics. This risk may be heightened in alcohol-dependent patients who already have a susceptibility to infection. Current literature frequently identifies an increased risk of pneumonia in patients with high alcohol use, owing to impaired host defence systems and chronic pulmonary inflammation [5]. In a systematic review, Samokhvalov et al. reported an eightfold increased risk of pneumonia in patients with alcohol use disorder [6]. Additionally, in mice models, Poole et al. demonstrated a link between chronic alcohol use and mechanical changes in the airways, which may contribute to the increased susceptibility to lung injury [7].

The severe pain caused by rib fractures can lead to hypoventilation and respiratory failure, which are significant causes of morbidity and mortality in thoracic trauma patients. However, it is commonly recognised that pain is under-treated in patients with a history of alcohol abuse [8]. Perry described a trend towards under medication in patients with chronic alcohol use due to clinician hesitancy around addiction and drug-seeking behaviour [9]. Furthermore, some authors found that patients with substance misuse disorders may even experience higher levels of pain after severe trauma [10]. Together, these factors may influence the likelihood of respiratory failure following rib fracture injuries in chronic alcoholics.

Identification of high-risk subgroups can aid early specialist review and help clinical decision-making regarding intensive care admissions. Despite the harmful physiological effects of chronic alcohol use and the high incidence of rib fractures in this demographic, no systematic review has previously been conducted to examine clinical outcomes in this patient group. Hence, the aim of this review is to collate the best available evidence to determine the effect of alcohol dependence on rib fracture outcomes. The primary outcome of interest is mortality and secondary outcome measures include in-hospital complications such as pneumonia, intensive care days and hospital length of stay.

This article was previously presented as a meeting abstract at the 2023 State of the Art Congress on 27 June 2023.

## Review

Methodology

This systematic review and meta-analysis was conducted in line with the preferred reporting items for systematic review and meta-analysis (PRISMA) guidelines [11]. A pre-registered and published protocol exists for this review on PROSPERO (CRD42022303709) [12].

Search Strategy

A systematic literature search was performed using the Embase, PubMed (Medline) and Web of Science databases with no date restrictions. The medical subject headings (MeSH) and keywords used in the search strategy combined the terms “rib fracture*”, “broken rib*”, “thorax injur*” and “thoracic trauma” with “alcohol*”, “substance abuse”, “substance misuse” and “heavy drink*”. To identify grey literature, the reference lists of included studies were manually reviewed and the clinicaltrials.gov website was searched for in-progress studies.

Inclusion and Exclusion Criteria

Studies were included in the review if they met the following criteria: 1) The study population included adult patients with one or more rib fractures; 2) Comparison of patients with a history of alcohol dependence against patients without alcohol dependence; 3) Outcome measures included mortality and relevant in-hospital complications; 4) Randomised control trials, quasi-randomised control trials and observational studies were included whilst case reports and editorials were excluded. Only articles available to view in English were included in the final review, as there was no funding available for translation services.

Alcohol Definition

Any terminology to confer alcohol dependence in the exposure group was accepted. Examples of these terms included chronic alcohol abuse, alcoholism, alcohol use disorder and alcohol addiction in line with code F10.2 from the International Statistical Classification of Diseases and Related Health Problems 10th Revision (ICD-10) [13]. Any study that measured acute intoxication or blood alcohol level on admission as the primary exposure was excluded, as these did not relate to a dependence syndrome.

Study Selection

Study selection was performed independently and in duplicate by two reviewers (FF and JO). Duplicates were removed after initial title and abstract screening. Full-text articles of relevant studies were then read to determine suitability for final inclusion against the review’s inclusion and exclusion criteria. Kappa agreement was 0.86 for full-text review and any disagreements were settled by the use of a third reviewer (AH).

Data Extraction

The following data were extracted from included studies and entered into a standardised table using Microsoft Excel (Microsoft Corporation, Redmond, WA): 1) Baseline characteristics of the studies, including sample size, setting and study design; 2) Definition of alcohol use in the exposure group and how this was ascertained; 3) Outcomes of interest; 4) Significant exclusion criteria. From each study, the control and exposure rate was extracted for mortality, pneumonia and acute respiratory distress syndrome (ARDS) and the odds ratio (OR) was calculated from these data if not already provided by the authors. If event rates were not available in meta-regression analyses, the crude data set was used. The median and interquartile range (IQR) was extracted for intensive care length of stay (LOS), hospital LOS and ventilator days.

Quality Assessment and Risk of Bias

The quality of included studies was assessed independently by two reviewers (FF, JO) using the Newcastle-Ottawa Scale. This is a tool recommended by Cochrane for non-randomised studies and involves a star system to assess three main aspects of the primary studies: selection of study groups, comparability and outcomes of interest [14]. A total of one star was awarded for each category with the exception of comparability, which received up to two stars. The results of this assessment were interpreted as follows: 8 or more, low risk of bias; 6-7, medium risk of bias; 5 or less, high risk of bias or unclear risk due to missing information.

Data Synthesis and Statistical Analysis

The OR for mortality, pneumonia and ARDS was extracted from each study as well as the median and IQR for intensive care LOS, hospital LOS and ventilator days. Ninety-five per cent (95%) confidence intervals were used to assess statistical significance, highlighted as a p-value <0.05.

A meta-analysis was performed using all three studies to pool results for mortality, pneumonia and ARDS. Review Manager (RevMan) software was used for the analysis and heterogeneity was assessed using the I2 statistic. The I2 result was interpreted based on suggested thresholds from the Cochrane Handbook [15]: 0-40% may not be important, 30-60% moderate heterogeneity, 50-90% substantial heterogeneity and 75-100% considerable heterogeneity. Owing to observed clinical heterogeneity between the primary studies, a random effects model was used in the meta-analyses.

Results

Literature Search and Study Characteristics

**Figure 1:**
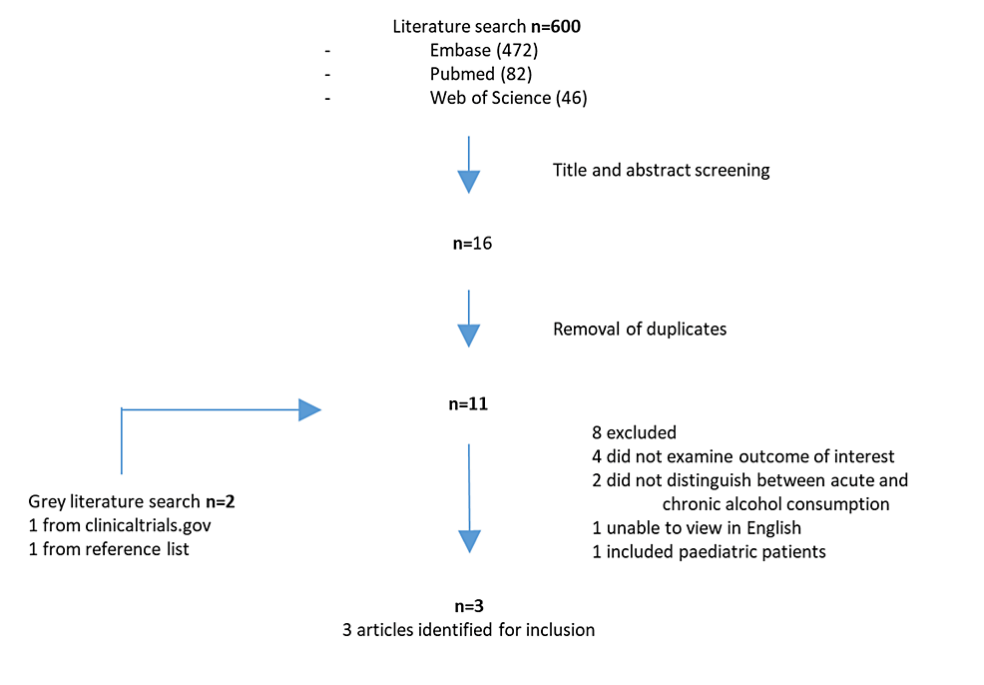
PRISMA flow diagram outlining the study selection process

The process for study inclusion is detailed in the flow diagram in Figure 1. A total of 600 studies were identified in the initial search (Embase = 472, PubMed = 82, Web of Science = 46). Reasons for study exclusion after full-text review were as follows: four did not examine the outcomes of interest; two did not distinguish between acute and chronic alcohol use; one included paediatric patients and one was not available to view in English. Overall, three studies were identified for final inclusion [16-18].

The baseline characteristics of the included studies are detailed in Table 1. Published dates of included studies ranged from 2019 to 2021, with a total of 41638 participants. All studies were conducted in North America and involved retrospective analysis of trauma databases. Two studies examined the same database during the same time period [16,17]. These studies had significantly larger sample sizes and compared equally sized cohorts. Two studies excluded patients with penetrating injuries [17,18] and one study [18] excluded patients who died within 24 hours of admission. All studies examined in-hospital complications, including mortality, pneumonia, ARDS, intensive care and hospital LOS and ventilator days. Two studies used 1:1 propensity matching to compare their cohorts to ensure no difference in age, gender, injury severity, number of rib fractures, smoking status, arrival vital signs or mechanism of injury between the two groups [16,17]. Gongola et al. controlled for age, gender, race and smoking status in a multi-variable regression model but did not control for any parameters in their initial analysis [18]. The crude data from this analysis were used in our review, as event rates were not provided in their regression model and some secondary outcomes were omitted.

**Table 1:** Baseline characteristics of included studies SD = standard deviation

Author and Year	Study design	Number of patients	Setting	Population	Definition of exposure	Measurement of exposure	Average age of control group (Years, SD)	Outcome or endpoint	Exclusion criteria
Vartanyan [16] 2019	Retrospective cohort	20120	Trauma Quality Improvement Program Database, North America	Adult patients with rib fractures	Chronic alcohol consumption	Not stated	47 +/- 12	In-hospital complications including mortality	Acute intoxication
Vartan [17] 2020	Retrospective cohort	19638	Trauma Quality Improvement Program Database, North America	Adult (≥18yrs) blunt trauma patients with rib fractures	Alcohol Use Disorder	Documentation in medical notes	53 +/- 22	In-hospital complications including mortality	Acute intoxication and penetrating injuries, the patient declared dead on arrival at the hospital
Gongola [18] 2021	Retrospective cohort	1880	Institutional Level 1 Trauma Centre Database, North America	Adult (≥18yrs) blunt trauma patients with rib fractures.	Alcohol Use Disorder	Documentation in medical notes	Not stated	In-hospital complications including mortality	Penetrating injuries, the patient died within 24 hours of admission

Risk of Bias

The results of the risk of bias assessment are presented in Table 2. No study provided detail on the length or adequacy of follow-up, and one study [16] failed to describe how alcohol dependence was ascertained in the exposed cohort. Overall, the scores ranged from five to seven with two studies graded as medium risk of bias and one study as high risk.

**Table 2:** Risk of bias assessment using the Newcastle-Ottowa Scale *Interpretation of scores: 8 or more – low risk of bias; 6-7 stars – medium risk of bias; 5 or less – high risk of bias or unclear risk due to missing information

Study	Selection					Outcome			Total number of stars*
	Representative of cohort	Selection of non-exposed cohort	Ascertainment of exposure	Outcome of interest	Comparability	Assessment	Length of follow-up	Adequacy of follow-up	
Vartanyan [16] 2019	*	*	-	*	*	*	-	-	5
Vartan [17] 2020	*	*	*	*	**	*	-	-	7
Gongola [18] 2021	*	*	*	*	*	*	-	-	6

Mortality

**Figure 2:**
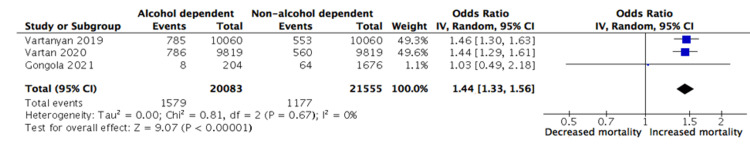
Meta-analysis of the association between alcohol dependency and mortality in patients with rib fractures Vartanyan [16], Vartan [17], Gongola [18] IV = inverse variance; CI = confidence interval

All studies measured in-hospital mortality, which allowed for comparison of the data in a meta-analysis. The mortality rate and OR for each study is displayed in Table 3. The meta-analysis of effect estimates for mortality is shown in Figure 2. The pooled OR was 1.44 (95% CI: 1.33-1.56) indicating a significantly increased risk of mortality associated with alcohol dependence. Statistical heterogeneity was low with an I2 result of 0% (p<0.01).

**Table 3:** Results for mortality in alcohol and non-alcohol-dependent patients OR = odds ratio

Study	Alcohol dependent	Non-alcohol dependent	OR	p-value
Vartanyan [16] 2019	7.8%	5.5%	1.46	<0.001
Vartan [17] 2020	8.0%	5.7%	1.44	<0.001
Gongola [18] 2021	3.9%	3.8%	1.03	0.942

Secondary Outcomes

**Figure 3:**
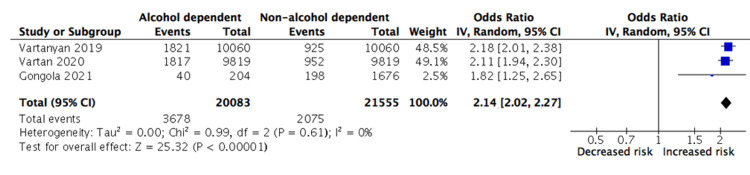
Meta-analysis of the association between alcohol dependency and pneumonia Vartanyan [16], Vartan [17], Gongola [18] IV = inverse variance; CI = confidence intervals

**Figure 4:**
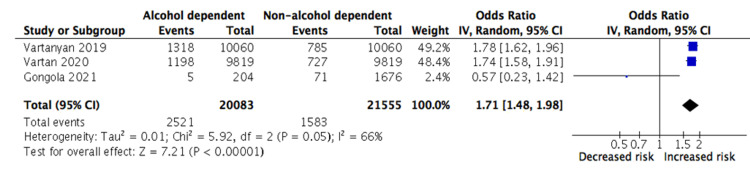
Meta-analysis of the association between alcohol dependency and ARDS Vartanyan [16], Vartan [17], Gongola [18] IV = inverse variance; CI = confidence intervals; ARDS = acute respiratory distress syndrome

The results for secondary outcomes are displayed in Table 4 and the meta-analyses for pneumonia and ARDS are demonstrated in Figures 3-4, respectively. Compared to non-alcohol dependent patients, there was a significantly increased risk of pneumonia (OR 2.14 {2.02-2.27}) and ARDS (OR 1.71 {1.48-1.98}) in the exposure group. The effect estimate for pneumonia was the strongest out of all the outcome measures and all studies were individually able to demonstrate a significant association with alcohol dependence. Conversely, there was substantial heterogeneity in the results for ARDS (I2 = 66%, p<0.01).

**Table 4:** Results for secondary outcomes in alcohol and non-alcohol-dependent patients OR = odds ratio

Outcome and Study	Alcohol dependent	Non-alcohol dependent	OR	p-value
Mortality (%)				
Vartanyan [16] 2019	7.8	5.5	1.46	<0.001
Vartan [17] 2020	8.0	5.7	1.44	<0.001
Gongola [18] 2021	3.9	3.8	1.03	0.942
Pneumonia (%)				
Vartanyan [16] 2019	18.1	9.2	2.18	<0.001
Vartan [17] 2020	18.5	9.7	2.11	<0.001
Gongola [18] 2021	19.6	11.8	1.82	<0.001
ARDS (%)				
Vartanyan [16] 2019	13.1	7.8	1.78	<0.001
Vartan [17] 2020	12.2	7.4	1.74	<0.001
Gongola [18] 2021	2.4	4.2	0.57	0.222
Intensive Care LOS (median {IQR})				
Vartanyan [16] 2019	5 {2-11}	4 {2-8}		0.015
Vartan [17] 2020	5 {2-11}	4 {2-9}		0.011
Gongola [18] 2021	3 {0-8}	0 {0-4}		<0.001
Ventilator days (median {IQR})				
Vartanyan [16] 2019	4 {2-10}	4 {2-9}		0.17
Vartan [17] 2020	4 {2-9}	4 {2-8}		0.168
Gongola [18] 2021	0 {0-3}	0 {0-2}		0.13
Hospital LOS (median {IQR})				
Vartanyan [16] 2019	7 {4-14}	6 {3-11}		0.025
Vartan [17] 2020	7 {4-12}	6 {3-10}		0.034
Gongola [18] 2021	7 {3-13}	5 {2-11}		<0.001

All studies demonstrated significantly increased intensive care and hospital LOS (p < 0.05) with alcohol dependence although there was no difference in ventilator days between the two groups.

Discussion

To our knowledge, this is the first systematic review to examine the effect of alcohol dependence on rib fracture outcomes. The results of this review show that alcohol dependence is associated with increased mortality, pneumonia, ARDS, intensive care LOS and hospital LOS following rib fractures compared to non-alcoholic patients. The findings highlight a need for careful monitoring and early intensive care review in this group of patients.

Mortality

The pooled OR for mortality demonstrates a significant association with alcohol dependence. However, Gongola et al. were unable to show statistical significance in their study with wide confidence intervals indicative of imprecise results [18]. This may be due to a smaller sample size, lack of control for injury parameters or exclusion of patients who died within 24 hours of admission. Furthermore, alcoholic patients in their study were older and more likely to smoke compared to the control group which may have confounded their results. However, is important to note that in their multi-variable regression model, which controlled for smoking status, age and gender, their results for mortality remained insignificant.

There is a paucity of previous literature investigating chronic alcoholism and mortality following trauma. A recent study examining patients with alcohol withdrawal syndrome, which is correlated to alcohol dependency, found no association with mortality after trauma [19]. Furthermore, in a subgroup analysis, Harfouche et al. found that alcohol use disorders were associated with lower mortality rates in severely injured patients [20]. Although both of these studies examined a broad trauma population, rather than rib fracture injuries specifically, they indicate that there is little consensus in the literature on how alcohol dependency influences mortality.

Additionally, all studies in this review examined in-hospital mortality however Dezman et al. examined 20-year mortality in trauma patients with substance misuse [21]. They found that alcohol use disorder was significantly associated with mortality after discharge, with injury as the most common cause of death. This suggests that whilst early mortality and alcohol dependence may be discrete, further research is needed to examine long-term outcomes in the patient population.

Secondary Outcomes

The effect estimates for pneumonia were consistent across all studies and the pooled OR showed the strongest association with alcohol dependence compared with other outcomes. This is in accordance with previous studies that have examined pulmonary complications in alcoholic trauma patients. Brasel et al. also demonstrated an increased risk of pneumonia following rib fractures in patients with a history of alcohol abuse and whilst their results were not significant, this could be attributed to the inclusion of paediatric patients in their study population [22].

Additionally, Marco et al. examined patients with rib fractures over a five-year period and found that alcohol consumption of one to five drinks per day was associated with a significantly increased risk of pneumonia [23]. Although their cohort does not represent a dependence syndrome, it supports a trend that chronic alcohol intake leads to worse outcomes following rib fractures. In a recent study by Carlson et al., pneumonia was also identified as a common and severe complication of alcohol withdrawal syndrome, suggesting that early management of withdrawal symptoms on admission to the hospital could reduce pulmonary complications [24]. Furthermore, early intubation was shown to reduce intensive care LOS in their study, although its impact on mortality and other adverse complications remains unclear.

The results for ARDS in this review showed substantial heterogeneity (I2 = 66%). Owing to the limited number of studies in the review, a subgroup analysis could not be performed to examine this further. In contrast to the other studies, Gongola et al. showed a decreased risk of ARDS in alcohol-dependent patients despite increased pneumonia and intensive care LOS [18]. One explanation for this discrepancy is that these authors examined a single institution in a rural American state and had lower incidences of ARDS in both cohorts, in contrast to the national databases used by the other studies. Owing to this heterogeneity in the meta-analysis, it is difficult to draw any meaningful conclusions about ARDS and further research is needed to examine this specific outcome.

Previous research, however, has been more consistent. In a large, five-year retrospective study, Tignanelli et al. found that chronic alcoholism was one of the strongest risk factors for the development of ARDS after direct thoracic injury alongside cardiopulmonary disease [25]. This is in addition to a recent systematic review, which also identified alcohol as a major risk factor for all-cause ARDS [26]. However, it is important to consider that all of the studies included in this review were observational in design and there was substantial heterogeneity in the definition of alcohol exposure, ranging from three alcoholic drinks per week to formal alcohol use disorders diagnosed with screening tools. Future research should be careful to distinguish alcohol dependence from general alcohol consumption in order to determine reliable associations.

The correlation between alcoholism and pulmonary disease is not fully understood, however, several possible explanations have been considered. Impaired mucociliary clearance of pathogens and a reduction in the recruitment and function of alveolar macrophages following chronic alcohol exposure have both been identified as causes of compromised host immunity [27,28]. This may be exacerbated following thoracic trauma when severe pain alters lung mechanics and weakens other host defences such as coughing. Furthermore, using inhaled isotopes and washouts in human subjects, Burnham et al. demonstrated an increased permeability of the alveolar-capillary barrier in alcohol-dependent patients [29]. This could explain the susceptibility to fluid accumulation and ARDS in this subgroup and importantly, the authors controlled for 13 patient factors, including smoking, lung disease and concurrent drug use, which further strengthens the evidence.

Overall, the risk of pneumonia and ARDS following rib fractures in alcohol-dependent patients suggests that careful monitoring of clinical symptoms and vital signs should be employed to manage and minimize these complications.

No study found a significant difference in ventilator days between the two groups despite significantly increased intensive care LOS in alcohol-dependent patients. One explanation for this may be the increased mortality in this cohort leading to a subsequent survival benefit in the control group. Alternatively, the discrepancy between these results may be attributed to other factors such as withdrawal symptoms, delirium or multi-organ failure, requiring longer intensive care stays.

Limitations

The review has some important limitations to consider. First, a low number of primary studies met the inclusion criteria for this review and all three studies were observational in design, which lowers the quality of evidence. Furthermore, the two studies with the greatest weighting in the meta-analyses examined the same study population over the same time period, which limits the generalisation of results.

Many studies examining alcohol use are subject to bias due to variations in how dependency syndromes are defined and diagnosed in the clinical setting. Identification often relies on taking a comprehensive social history alongside the use of patient scoring systems which are subjective. No study in this review was able to provide information on how the diagnoses of alcohol use disorder were made in their exposure cohorts and, as all studies were retrospective, there is a further risk of measurement bias owing to the number of patients who may have been excluded due to incorrect coding or poor documentation.

Reporting bias was minimized by an examination of reference lists for grey literature and searching the clinicaltrials.gov website for in-progress studies. However, a funnel plot could not be used to formally assess publication bias due to the low number of studies included in the final review.

Although all studies discussed the risk of confounding bias from variables such as age and smoking status, co-dependency on other drugs was not controlled for in any study. This may be relevant to future research considering that alcohol and substance misuse disorders commonly co-occur and opioid addiction has been shown to independently worsen outcomes following trauma [30]. Furthermore, owing to this correlation, it may be important to measure long-term, community outcomes in this demographic such as the risk of analgesic dependency following discharge after rib fracture injuries.

Whilst there is limited primary research examining rib fracture outcomes in this cohort of patients, the results of this review support a large body of previous literature identifying alcohol dependence as a risk factor for pneumonia, ARDS and poor outcomes after trauma. Clinicians assessing these patients in the emergency department should therefore consider early referral to intensive care and ensure timely assessment and management of withdrawal symptoms to further reduce the risk of pulmonary complications. Additionally, current rib fracture scoring systems could be modified to include alcohol dependency as a high-risk factor to aid clinicians in this decision process and improve outcomes for these patients.

## Conclusions

The results of this review support previous research associating alcohol dependence with poor outcomes following thoracic trauma. Specifically, these patients have an increased risk of mortality, ARDS and pneumonia, and experience longer intensive care and hospital stays following rib fractures. Early intensive care review and modification of rib fracture scoring systems to include this high-risk group could help minimize adverse complications. However, these results should be interpreted with caution owing to the limited number of primary studies and low quality of evidence. Future research should ensure that confounding factors, such as the number of rib fractures, smoking status and co-dependency on other drugs, are controlled for. Overall, whilst there is a need for further high-quality primary research to understand the relationship between alcohol dependence and rib fracture outcomes, the results of this review indicate a rationale for careful monitoring and a low threshold for intensive care admission in this patient population.
